#  Evidence for a Specific Diabetic Cardiomyopathy: An Observational Retrospective Echocardiographic Study in 656 Asymptomatic Type 2 Diabetic Patients

**DOI:** 10.1155/2015/743503

**Published:** 2015-05-13

**Authors:** Isabelle Pham, Emmanuel Cosson, Minh Tuan Nguyen, Isabela Banu, Isabelle Genevois, Patricia Poignard, Paul Valensi

**Affiliations:** ^1^Department of Physiology, AP-HP, Jean Verdier Hospital, 93 143 Bondy, France; ^2^Sorbonne Paris Cité-Université Paris 13, UFR SMBH, EA 23-63, 93 017 Bobigny, France; ^3^AP-HP, Jean Verdier Hospital, Department of Endocrinology-Diabetology-Nutrition, Université Paris 13, CRNH-IdF, CINFO, 93 143 Bondy, France; ^4^Sorbonne Paris Cité-Université Paris 13, UMR U1153 Inserm/U1125 Inra/Cnam, 93 017 Bobigny, France

## Abstract

*Aim*. Our aim was to assess the prevalence of subclinical diabetic cardiomyopathy, occurring among diabetic patients without hypertension or coronary artery disease (CAD). *Methods*. 656 asymptomatic patients with type 2 diabetes for 14 ± 8 years (359 men, 59.7 ± 8.7 years old, HbA1c 8.7 ± 2.1%) and at least one cardiovascular risk factor had a cardiac echography at rest, a stress cardiac scintigraphy to screen for silent myocardial ischemia (SMI), and, in case of SMI, a coronary angiography to screen for silent CAD. *Results*. SMI was diagnosed in 206 patients, and 71 of them had CAD. In the 157 patients without hypertension or CAD, left ventricular hypertrophy (LVH: 24.1%) was the most frequent abnormality, followed by left ventricular dilation (8.6%), hypokinesia (5.3%), and systolic dysfunction (3.8%). SMI was independently associated with hypokinesia (odds ratio 14.7 [2.7–81.7], *p* < 0.01) and systolic dysfunction (OR 114.6 [1.7–7907], *p* < 0.01), while HbA1c (OR 1.9 [1.1–3.2], *p* < 0.05) and body mass index (OR 1.6 [1.1–2.4], *p* < 0.05) were associated with systolic dysfunction. LVH was more prevalent among hypertensive patients and hypokinesia in the patients with CAD. *Conclusion*. In asymptomatic type 2 diabetic patients, diabetic cardiomyopathy is highly prevalent and is predominantly characterized by LVH. SMI, obesity, and poor glycemic control contribute to structural and functional LV abnormalities.

## 1. Introduction

Myocardial impairment in diabetes mellitus is due to multiple pathophysiological pathways involving myocardial ischemia and/or coronary artery disease (CAD) and hypertension, which are usual in type 2 diabetes. When thosefactors are excluded, myocardial impairment is considered to be specific to diabetes, defining diabetic cardiomyopathy [1]. Cardiac function abnormalities on echography have been extensively described but hypertension and/or CAD have not been always excluded in the cohorts studied. Previous large studies have shown that left ventricle (LV) structure abnormalities including hypertrophy and/or concentric remodelling, diastolic filling, and relaxation alterations are often present in early stages of diabetes mellitus, before symptomatic heart failure [2–6]. However, these findings were not specific to diabetic cardiomyopathy as ischemic status was not always assessed in these studies. This is an important issue as silent myocardial ischemia (SMI) is a common complication in diabetes with additional risk factors. Thus, we have previously reported that considering LV abnormalities including hypertrophy, systolic dysfunction, or hypokinesia detected by cardiac echography in asymptomatic type 2 diabetic patients significantly improves CAD prediction [7]. Recently, other studies using tissue Doppler or strain rate parameters have also shown early and subclinical systolic impairment [8–11] and systolic dysfunction may precede diastolic dysfunction [12]. In those latter papers [8–11], although myocardial ischemia was excluded, some patients had hypertension and the number of patients was much smaller than in former papers [2–6]. Cardiac echographic features may be associated with diabetes and most often with older age, male gender, higher body mass index (BMI), hypertension, renal dysfunction, or other metabolic parameters such as dyslipidemia [2, 3, 12, 13]. Although several recent studies have confirmed higher prevalence and severity of heart failure in diabetic than in nondiabetic patients [14–16], the specificity and the importance of diabetic cardiomyopathy are still debated [17, 18].

Here, we report the prevalence of LV mass and function abnormalities on echocardiography and their determinants in a retrospective series of 656 asymptomatic diabetic patients referred for cardiac and vascular complications screening. Patients were then sorted out according to the presence of hypertension and silent CAD and data on the group without CAD and without hypertension were analysed in order to characterize the determinants of diabetic cardiomyopathy in the absence of confounding factors.

## 2. Methods

### 2.1. Patients

We collected the data of type 2 diabetic inpatients of the Department of Endocrinology-Diabetology-Nutrition, Jean Verdier Hospital (Bondy, France), between 1991 and 2008. Criteria for inclusion in this study were normal 12-lead resting ECG and the presence of at least one of the following additional cardiovascular risk factors: dyslipidemia (serum total cholesterol > 6.5 mmol/L, or triglycerides > 2.3 mmol/L, or lipid-lowering treatment), hypertension (blood pressure ≥ 140/90 mmHg or antihypertensive treatment), smoking, microalbuminuria (albumin excretion rate > 30 mg/day on at least two assessments), family history of premature CAD (before the age of 60 in first-degree relatives), proximal peripheral (stenosis ≥ 50% on femoral or popliteal arteries), or carotid (stenosis ≥ 50% on extracranial carotid artery) occlusive arterial disease detected by ultrasound examination. Criteria for noninclusion were history of myocardial infarction or angina pectoris, congenital heart disease, known cardiomyopathy or valve diseases, and ECG ischemic abnormalities. Diabetic retinopathy was diagnosed if at least one microaneurysm or hemorrhage was found on eye fundus examination. The diagnosis of peripheral neuropathy was based on the presence of any two or more of the following: neuropathic symptoms, decreased distal sensation, or decreased or absent ankle reflexes. Each patient enrolled in this study gave oral informed consent in accordance with the European directives.

### 2.2. Cardiac Investigations

#### 2.2.1. Cardiac Transthoracic Echocardiography

Rest cardiac transthoracic echocardiography was performed on Acuson XP128 before 2004 and Sequoia C512 (Siemens, Erlangen, Germany) after 2004. Two-dimensional images were acquired on parasternal and apical views, time-motion images were acquired on one parasternal view, and pulsed-wave Doppler was used with a sample volume of 2 mm and a sweep speed of 100 mm/s. Measurements and calculations were done according tothe recommendations of the American Society of Echocardiography [19]. Patients with aortic stenosis were not included in the study. LV volumes were measured in a 4-chamber and 2-chamber apical view. LV systolic function was assessed by the ejection fraction that was calculated with Simpson's method. LV diameters and wall thickness were measured in a parasternal long-axis view using the M-mode that also allowed the calculation of the LV ejection fraction (Teicholz formula) in the absence of segmental hypokinesia. LV mass was calculated according to American Society of Echocardiography's formula and normalized with the body surface area.

#### 2.2.2. Screening for Silent Myocardial Ischemia and CAD

Each patient underwent a thallium-201 myocardial scintigraphy after an ECG stress test and/or a pharmacological stress test (dipyridamole injection). The ECG stress test protocol was previously reported [20]. Briefly, the ECG stress test was performed according to the modified Bruce protocol. Single photon emission computed tomography (SPECT) gated acquisition was carried out with early images, that is, peak exercise or 4 min after a dipyridamole injection if the exercise test was not contributive (when the patient was unable to reach 85% of the maximal predicted heart rate (220-age) or when the ECG was indeterminate) and with delayed images (4.0 ± 0.5 hours later). The perfusion pattern (normal or showing stable or reversible defects) and ECG data (considered as positive if 1 mm flat or down-sloping ST segment occurred at 0.08 s after the J point with or without angina pectoris) were assessed by a nuclear medicine physician and a cardiologist, respectively, who were unaware of the clinical or ECG data and of the imaging data. SMI was defined as abnormal results of the ECG stress test and/or myocardial scintigraphy imaging.

Patients with SMI underwent a selective coronary angiography within 2 months after the noninvasive investigation. CAD was defined as a ≥70% narrowing of the luminal diameter in either the left anterior descending artery, the circumflex artery, the well-developed marginal vessel, or the right coronary artery or a ≥50% diameter narrowing of the left main coronary artery. The percentage of narrowing was visually determined by the consensus of two experienced investigators. In case of discrepancy between the two investigators, automatic quantification was used.

### 2.3. Biological Measurements

The following measurements were performed at the time of screening for SMI: HbA_1c_ (Dimension Technology, Siemens Healthcare Diagnosis Inc., Newark, USA), fasting plasma glucose (measured with the glucose oxidase method, colorimetry, Kone Optima, Thermolab System), serum total cholesterol, HDL cholesterol and triglycerides (enzymatic colorimetry, Hitachi 912, Roche Diagnostic, Meylan, France), creatininemia (colorimetry, Kone Optima, Thermolab System, Paris La Défense, France), and the 24 h urinary albumin excretion rate (laser immunonephelometry, BN100, Dade-Behring, Paris, France). LDL cholesterol was calculated according to the Friedwald formula and creatinine clearance with Cockroft's formula.

### 2.4. Statistical Analyses

Data are summarized as means ± SD for continuous variables, number of cases, and percentages for qualitative variables. Differences between groups were assessed by ANOVA tests or by Mann Whitney test for continuous variables. The Chi square test or the exact Fisher test was used for qualitative variables. To determine the significant independent predictors of echocardiographic abnormalities, stepwise logistic regression analyses were performed with the variables/parameters that were associated with each echocardiographic feature in univariate analysis with *p* < 0.1. Statistical analyses were carried out using SPSS software (SPSS, Chicago, IL). The 0.05 probability level was used for statistical significance.

## 3. Results 

### 3.1. Patients Characteristics

A total of 656 type 2 diabetic patients having been screened for SMI with myocardial scintigraphy and having interpretable echographic data were included. Of them, 483 (73.6%) had hypertension and SMI was diagnosed in 206 (31.4%) patients. Out of these 206 patients, 189 subsequently underwent a coronary angiography. Among them, 71 (11.1%) had significant CAD. Thus, 157 patients had neither CAD nor hypertension (Table 1). Compared to patients who had hypertension or CAD or both, those without hypertension and CAD were younger and had shorter diabetes duration, lower BMI, and higher total and LDL cholesterol levels, maybe related to a lower prevalence of lipid-lowering treatment (odds ratio (OR) 0.49 [95% confidence interval 0.33–0.71]) (Table 1). By definition, they were less likely to have antihypertensive treatment and SMI. Additionally, they had a lower prevalence of retinopathy (OR 0.52 [0.35–0.78]) and nephropathy (OR 0.41 [0.27–0.62]).

### 3.2. Echographic Abnormalities

LV hypertrophy, dilation, systolic dysfunction, and hypokinesia could be assessed in 566, 584, 548, and 603 patients, respectively. Missing data were due to poor echogenicity or segmental hypokinesia, which did not allow a reliable evaluation of the ejection fraction with Teicholz method. Echocardiographic examination showed LV hypertrophy in 181 patients (31.9%), LV dilation in 43 (7.3%), systolic dysfunction in 21 (3.8%), and hypokinesia in 49 (8.1%) (Table 2).

**Figure 1:**
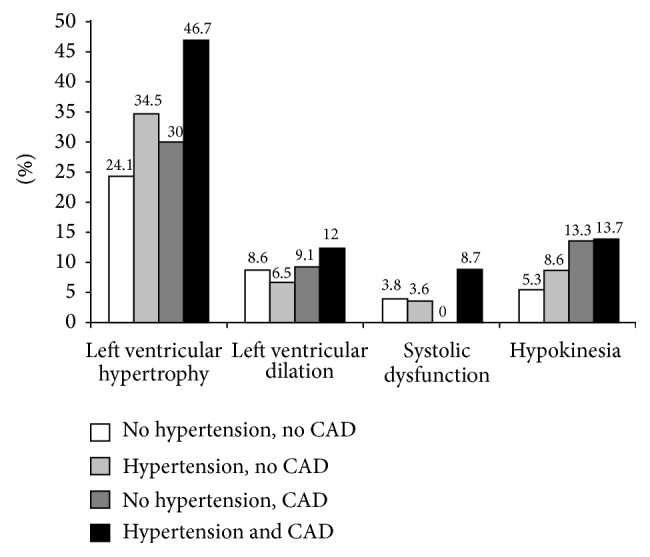
Prevalence of cardiac echographic disorders according to the presence of coronary artery disease (CAD) and/or hypertension.

Figure 1 shows the prevalence of echographic disorders in four exclusive groups of patients defined according to the presence or the absence of CAD and/or hypertension. Kinds of prevalence of LV hypertrophy, dilation, systolic dysfunction, and hypokinesia were the highest in patients with CAD and hypertension and were, respectively, 46.7, 12, 8.7, and 13.7%. In patients without CAD and hypertension, the most frequent echography abnormality was LV hypertrophy (24%).

### 3.3. Determinants of Echographic Abnormalities in the Patients without CAD or Hypertension

LV hypertrophy was associated with higher BMI and body weight and lower LDL cholesterol levels (Table 3). In multivariate analysis taking into account both parameters, only LDL cholesterol levels were independently associated with LV hypertrophy (OR 0.58 [0.35–0.98], *p* < 0.05).

Table 3 shows that LV dilation was associated with a lower BMI and a higher creatinine clearance, with a trend for higher HDL cholesterol levels. A multivariate analysis taking into account these parameters found that only BMI was independently associated with LV dilation (OR 0.65 [0.45–0.93], *p* < 0.05).

Systolic dysfunction was associated with higher BMI and HbA1c levels and with SMI (OR 8.5 [1.3–54.5]). Multivariate analysis showed that the three parameters were independently associated with systolic dysfunction: BMI (OR 1.6 [1.1–2.3], *p* < 0.05), HbA1c (OR 1.9 [1.1–3.2], *p* < 0.05), and SMI (OR 95 [2–5162], *p* < 0.05).

Hypokinesia was associated with SMI (OR 10.8 [2.4–48.9]), with a trend for an association with peripheral occlusive arterial disease and lipid-lowering treatment. Multivariate analysis showed that SMI (OR 14.2 [2.5–81.2], *p* < 0.01) and peripheral occlusive arterial disease (OR 9.7 [1.01–93.5], *p* < 0.05) were independently associated with hypokinesia.

## 4. Discussion

In the present study, we investigated the echographic abnormalities in a large series of diabetic patients without cardiac history or symptoms. In order to provide additional support to the cardiomyopathy entity, we carefully assessed all patients for SMI and subsequently for CAD in those with SMI. We confirm that LV hypertrophy was the most frequent echocardiographic abnormality in type 2 diabetic patients without CAD and hypertension, whereas systolic parameters were impaired in less than 10% of the patients. In the diabetic patients with both, these abnormalities were 1.5- to 3-fold more frequent than in those without hypertension and CAD. In the patients without CAD or hypertension, that is, those potentially with echographic abnormalities likely to be related to diabetic cardiomyopathy, systolic dysfunction was significantly associated with glucose levels, SMI, and BMI while hypokinesia was only related to SMI. Finally, LV hypertrophy was associated with LDL cholesterol and LV dilation with BMI.

Although the first papers on diabetic cardiomyopathy by Rubler et al. and then Regan et al. clearly defined this entity in diabetic patients without hypertension and without CAD, there are still some debates on a specific diabetic cardiac impairment [1, 21]. Diabetic cardiomyopathy is difficult to assess, because of confounding factors (mostly hypertension) and the heterogeneity of the echocardiographic parameters used to evaluate LV function. Notably, systolic and diastolic functions, which were evaluated by conventional parameters in previous large studies, are nowadays assessed by parameters derived from tissue Doppler imaging and/or strain rate but in smaller populations.

The initial large studies on cardiac function found that LV hypertrophy and diastolic impairment evaluated by mitral inflow study were more frequent in patients with type 2 diabetes than in patients with impaired glucose tolerance or patients with and without hypertension [2–4, 6]. There was a significant association between glycemic control and increased LV mass [3, 4]. Moreover, patients with a medical history of heart failure or CAD were excluded from the study but whether they had silent myocardial ischemia and asymptomatic CAD was unknown [2].

Prevalence of systolic dysfunction in asymptomatic patients is not often studied and as in our results concerns less than 10% of the diabetic population. Dawson et al. found systolic dysfunction in 4% of 500 diabetic patients, among whom 61% had hypertension and 16% ischemic heart disease or stroke [6]. Somaratne et al. recently found LV hypertrophy, systolic dysfunction, and hypokinesia, respectively, in 56, 4, and 6% of 294 asymptomatic type 2 diabetic patients without history or symptom of CAD [22]. However, 60% of these patients had hypertension and LV hypertrophy was reported only in 32% of the diabetic nonhypertensive patients. Other features of cardiac echography were not reported for this subgroup and determinants of these abnormalities were not assessed. Other papers have also shown early systolic impairment detected by tissue Doppler or speckle tracking, without alteration of conventional systolic function parameters in small series of patients compared to matched control subjects [8–11]. However, although myocardial ischemia was excluded, hypertension was not an exclusion criterion [9, 11] or affected the majority of the patients [10]. More interestingly, Ernande et al. reported systolic longitudinal and radial dysfunction without diastolic dysfunction in 28% of 114 diabetic patients without hypertension and myocardial ischemia. This is the first study to find a systolic impairment as the earliest marker of cardiac injury before diastolic impairment which is usually considered as the first alteration in diabetic cardiomyopathy [12].

In our data, a high BMI was consistently associated with LV hypertrophy, LV dilation, and dysfunction, while poor glycaemic control (high HbA1c) was associated only with systolic dysfunction. This is consistent with the role of obesity which affected most of these patients and which has been shown to induce structural and myocardial disorders independently of diabetes [23–25]. Only recent studies evaluating systolic function by strain rate in diabetic patients and matched control subjects have found a significant link between diabetes and early alteration in systolic function (in the absence of change in ejection fraction or other conventional parameters) [9–12]. The pathophysiology of diabetic cardiomyopathy is not yet fully understood and its development is likely to be multifactorial. Although chronic hyperglycemia is thought to play a central role, multiple complex mechanisms and interplay of many molecular and metabolic events within the myocardium and plasma contribute to the pathogenesis. The significant association with BMI is in agreement with the involvement of metabolic impairment related to insulin resistance and free fatty acids in the pathophysiology of diabetic cardiomyopathy [26, 27]. Microvascular disease, by inducing focal ischemia and fibrosis associated with the metabolic factors, may be involved in the myocardial changes. Interestingly, we also found a significant link between SMI and hypokinesia and systolic dysfunction. As there was no significant CAD in these patients, the presence of SMI may indicate a possible microvascular contribution to the development of diabetes-associated cardiac dysfunction and diabetic cardiomyopathy. Consistent with this hypothesis we previously reported a reduction of the coronary microcirculation dilation in response to a cold-pressor test in diabetic patients with SMI but without CAD on angiography [28]. Lastly structural changes in myocardium and autonomic dysfunction are probably involved [26, 27].

### 4.1. Study Limitations

The data collected in this paper concern a cohort that was established since the 1990s, implying that only conventional measurements were complete for the whole population. For example, the Teicholz method was the only available method to determine ejection fraction for some patients, especially before year 2000, although it is not recommended any more. Furthermore, complete diastolic parameters including tissue Doppler imaging measurements are lacking. At the present time, myocardial function examination needs the regional myocardial function assessment. The low prevalence of LV dilation, systolic dysfunction, or focal hypokinesia may have also weakened the statistical analysis. We have only explored inpatients with at least one risk factor in addition to diabetes; therefore, the results are not necessarily generalizable to the diabetic population. Only patients with an abnormal myocardial scintiscan underwent a coronary angiography, and some false negative results might have prevented us from diagnosing asymptomatic CAD. However, it appears unethical to perform a coronary angiography in asymptomatic patients without myocardial ischemia.

## 5. Conclusion

This carefully designed study included a large population of asymptomatic type 2 diabetic patients without hypertension and CAD and shows that cardiomyopathy is highly prevalent and predominantly characterized by LVH. The mechanisms involved in LV hypertrophy need further clarification. Higher HbA1c levels were only associated with systolic dysfunction, while SMI was linked to systolic dysfunction and hypokinesia suggesting a role played by microvascular impairment. Overweight is likely to play a role in myocardial systolic disorders.

Further large studies using conventional parameters with diastolic parameters as defined by the most recent recommendation [29] and subclinical markers of LV function (strain, strain rate, and torsion) are needed to improve the comprehension of the pathophysiology of diabetic cardiomyopathy but also for the risk stratification in asymptomatic patients.

## Figures and Tables

**Table 1:** Characteristics of the total cohort of type 2 diabetic patients, of those without hypertension and coronary artery disease, and of those with hypertension or coronary artery disease.

	Total	Without hypertension and CAD	With hypertension or CAD	*p*
	*n* = 656	*n* = 157	*n* = 482^∗^	
Age, years	59.7 ± 8.7	56.7 ± 8.6	60.6 ± 8.6	<0.0001
Gender (male/female)	359/297	95/62	257/225	NS
Diabetes duration, years	13.7 ± 7.7	11.9 ± 7.2	14.2 ± 7.8	0.001
Body mass index, kg/m^2^	30.3 ± 6.1	28.5 ± 5.5	31.0 ± 6.2	<0.0001
Body weight (kg)	82.6 ± 16.8	78.0 ± 15.9	84.1 ± 16.8	<0.0001
HbA1c, %	8.7 ± 2.1	8.7 ± 2.0	8.7 ± 2.2	NS
Retinopathy (%)	242 (38.2)	42 (27.1)	192 (41.6)	0.001
Nephropathy (%)	256 (39.2)	38 (24.2)	210 (43.8)	<0.0001
Urinary albumin excretion rate, mg/day	161 ± 505	55 ± 205	194 ± 567	<0.05
Creatinine clearance (mL/min)	83 ± 24	88 ± 19	81 ± 25	<0.01
Peripheral occlusive arterial disease (%)	64 (10.1)	10 (6.5)	53 (11.4)	0.09
Peripheral neuropathy (%)	292 (45.0)	65 (41.4)	213 (44.8)	NS
Silent myocardial ischemia (%)	206 (31.4)	26 (16.6)	163 (33.8)	<0.0001
CAD (%)	71 (11.1)	/	71 (14.7)	/
Hypertension (%)	483 (73.6)	/	467 (96.9)	/
Antihypertensive therapy (%)	437 (67.8)	/	423 (89.6)	/
Systolic blood pressure, mmHg	135 ± 19	125 ± 11	138 ± 20	<0.0001
Diastolic blood pressure, mmHg	75 ± 11	71 ± 9	76 ± 11	<0.0001
Dyslipidemia (%)	445 (69.9)	97 (64.2)	334 (71.2)	0.127
Lipid-lowering treatment, %	285 (44.4)	48 (31.2)	228 (48.3)	<0.0001
Total cholesterol, mmol/L	5.1 ± 1.2	5.2 ± 1.0	5.0 ± 1.2	<0.05
HDL cholesterol, mmol/L	1.2 ± 0.5	1.2 ± 0.4	1.2 ± 0.5	NS
LDL cholesterol, mmol/L	3.0 ± 1.0	3.2 ± 0.9	2.9 ± 1.0	<0.05
Triglycerides, mmol/L	1.9 ± 1.2	1.9 ± 1.1	1.9 ± 1.2	NS
Smoking (%)	142 (21.8)	37 (23.7)	104 (21.7)	NS
Familial history of premature CAD (%)	74 (11.5)	16 (10.5)	54 (11.4)	NS

^∗^17 patients had silent myocardial ischemia but did not undergo a coronary angiography.

CAD: coronary artery disease; NS: nonsignificant (*p* > 0.1).

Data are mean ± SD or *n* (%).

**Table 2:** Echocardiographic findings of the total cohort of type 2 diabetic patients, of those without hypertension and coronary artery disease, and of those with hypertension or coronary artery disease.

	Total	Without hypertension and CAD *n* = 157	With hypertension or CAD *n* = 482	*p*
LV systolic diameter, mm	29.5 ± 5.1	29.0 ± 5.3	29.7 ± 5.1	NS
LV diastolic diameter, mm	47.5 ± 5.3	46.7 ± 5.6	47.8 ± 5.2	<0.05
LV mass, g/m^2^	99.7 ± 27.4	90.8 ± 23.4	102.6 ± 28.0	<0.0001
Ejection fraction, %	67.1 ± 9.0	67.4 ± 9.4	67.1 ± 8.8	NS
Left ventricular hypertrophy (%)	181 (31.9)	33 (24.1)	148 (35.7)	<0.05
Left ventricular dilatation (%)	43 (7.3)	12 (8.6)	31 (7.2)	NS
Systolic dysfunction (%)	21 (3.8)	5 (3.8)	16 (4.0)	NS
Hypokinesia (%)	49 (8.1)	8 (5.3)	41 (9.4)	NS

CAD: coronary artery disease; LV: left ventricular; and NS: nonsignificant (*p* > 0.1).

Data are mean ± SD or *n* (%).

**Table 3:** Characteristics of the patients without coronary artery disease and hypertension.

	No LV hypertrophy	LV hypertrophy	*p*	No LV dilation	LV dilation	*p*	No systolic dysfunction	Systolic dysfunction	*p*	No hypokinesia	Hypokinesia	*p*
	*n* = 104	*n* = 33		*n* = 128	*n* = 12		*n* = 127	*n* = 5		*n* = 142	*n* = 8	
Age, years	56.7 ± 8.9	58.6 ± 7.5	NS	57.0 ± 8.8	57.8 ± 6.0	NS	57.0 ± 8.2	53.8 ± 10.8	NS	56.7 ± 8.5	58.8 ± 8.8	NS
Gender (male/female)	60/44	24/9	NS	77/51	8/4	NS	80/47	2/3	NS	87/55	4/4	NS
Diabetes duration, years	12.0 ± 7.0	12.4 ± 8.8	NS	11.8 ± 7.4	14.3 ± 7.9	NS	12.0 ± 7.3	10.2 ± 8.6	NS	11.7 ± 7.1	12.1 ± 6.7	NS
Body mass index, kg/m^2^	27.5 ± 4.3	29.5 ± 5.6	<0.05	28.7 ± 5.1	23.9 ± 2.5	<0.01	28.0 ± 4.6	32.4 ± 6.2	<0.05	28.6 ± 5.7	26.5 ± 2.5	NS
HbA1c, %	8.8 ± 2.1	8.6 ± 1.8	NS	8.8 ± 2.0	8.2 ± 2.2	NS	8.7 ± 2.0	11.0 ± 1.7	<0.05	8.8 ± 2.0	8.7 ± 2.4	NS
Retinopathy (%)	26 (25.5)	10 (30.3)	NS	34 (27.0)	5 (41.7)	NS	33 (26.2)	1 (20.0)	NS	36 (25.4)	3 (42.9)	NS
Nephropathy (%)	27 (26.0)	8 (24.2)	NS	31 (24.2)	4 (33.3)	NS	31 (24.4)	0 (0)	NS	34 (23.9)	0 (0)	NS
UAER, mg/24 hours	68.0 ± 251.0	36.0 ± 53.5	NS	61.9 ± 224.5	25.3 ± 21.4	NS	54.8 ± 220.2	12.9 ± 9.0	NS	50.7 ± 208.9	10.7 ± 6.9	NS
Creatinine clearance (mL/min)	88.0 ± 18.2	87.8 ± 20.2	NS	86.4 ± 17.7	100.1 ± 22.3	<0.05	89.0 ± 18.2	76.5 ± 18.2	NS	89.4 ± 18.8	82.8 ± 16.7	NS
POAD (%)	7 (6.8)	2 (6.7)	NS	8 (6.4)	1 (9.1)	NS	9 (7.3)	0 (0)	NS	7 (5.1)	2 (25.0)	0.08
Peripheral neuropathy (%)	42 (40.4)	15 (45.5)	NS	51 (39.8)	6 (50.0)	NS	55 (43.3)	1 (20.0)	NS	58 (40.8)	5 (62.5)	NS
Silent myocardial ischemia (%)	20 (19.2)	4 (12.1)	NS	23 (18.0)	1 (8.3)	NS	19 (15.0)	3 (60.0)	<0.05	19 (13.4)	5 (62.5)	<0.01
Antihypertensive therapy (%)	/	/	/	/	/	/	/	/	/	/	/	/
Systolic blood pressure, mmHg	124.6 ± 11.4	127.3 ± 9.6	NS	125.0 ± 11.3	125.3 ± 6.4	NS	125.4 ± 11.0	121.6 ± 12.5	NS	124.6 ± 11.6	121.8 ± 6.9	NS
Diastolic blood pressure, mmHg	71.6 ± 9.1	72.0 ± 8.5	NS	71.6 ± 8.9	73.4 ± 7.7	NS	71.6 ± 9.0	69.8 ± 6.3	NS	71.3 ± 9.0	70.3 ± 6.8	NS
Pulse pressure, mmHg	53.0 ± 11.6	55.3 ± 10.5	NS	53.4 ± 11.6	51.8 ± 8.9	NS	53.7 ± 11.6	51.8 ± 8.9	NS	53.3 ± 11.5	51.5 ± 7.9	NS
Dyslipidemia (%)	63 (63.0)	22 (71.0)	NS	82 (66.7)	6 (54.5)	NS	79 (65.3)	3 (60.0)	NS	86 (63.2)	6 (75.0)	NS
Lipid-lowering treatment, %	31 (30.4)	11 (33.3)	NS	41 (32.5)	2 (18.2)	NS	40 (31.5)	1 (20.0)	NS	41 (28.9)	5 (62.5)	0.06
Total cholesterol, mmol/L	5.3 ± 1.0	5.1 ± 1.0	NS	5.2 ± 1.0	5.1 ± 0.7	NS	5.2 ± 1.0	5.1 ± 1.2	NS	5.2 ± 1.0	5.3 ± 1.1	NS
HDL cholesterol, mmol/L	1.2 ± 0.4	1.2 ± 0.4	NS	1.2 ± 0.4	1.4 ± 0.5	0.08	1.2 ± 0.4	1.3 ± 0.4	NS	1.2 ± 0.4	1.1 ± 0.3	NS
LDL cholesterol, mmol/L	3.3 ± 0.9	2.9 ± 0.9	<0.05	3.2 ± 0.9	3.0 ± 0.9	NS	3.2 ± 0.9	2.9 ± 0.9	NS	3.1 ± 0.9	3.4 ± 1.1	NS
Triglycerides, mmol/L	1.9 ± 1.1	2.0 ± 1.3	NS	2.0 ± 1.1	1.4 ± 0.7	NS	1.9 ± 1.2	1.9 ± 0.6	NS	2.0 ± 1.2	1.5 ± 0.5	NS
Smoking (%)	27 (26.2)	8 (24.2)	NS	33 (26.0)	3 (25.0)	NS	31 (24.6)	2 (40.0)	NS	32 (22.7)	3 (37.5)	NS
Familial history of premature CAD (%)	11 (10.9)	3 (9.1)	NS	13 (10.3)	1 (9.1)	NS	12 (9.7)	1 (20.0)	NS	13 (9.4)	1 (12.5)	NS

CAD: coronary artery disease, LV: left ventricle, POAD: peripheral occlusive arterial disease, UAER: urinary albumin excretion rate, and NS: nonsignificant (*p* > 0.10).

## References

[1] Rubler S., Dlugash J., Yuceoglu Y. Z., Kumral T., Branwood A. W., Grishman A. (1972). New type of cardiomyopathy associated with diabetic glomerulosclerosis. *The American Journal of Cardiology*.

[2] Liu J. E., Palmieri V., Roman M. J. (2001). The impact of diabetes on left ventricular filling pattern in normotensive and hypertensive adults: the strong heart study. *Journal of the American College of Cardiology*.

[3] Galderisi M., Anderson K. M., Wilson P. W. F., Levy D. (1991). Echocardiographic evidence for the existence of a distinct diabetic cardiomyopathy (the Framingham Heart Study). *The American Journal of Cardiology*.

[4] Devereux R. B., Roman M. J., Paranicas M. (2000). Impact of diabetes on cardiac structure and function: the Strong Heart Study. *Circulation*.

[5] Srivastava P. M., Calafiore P., MacIsaac R. K. (2008). Prevalence and predictors of cardiac hypertrophy and dysfunction in patients with Type 2 diabetes. *Clinical Science*.

[6] Dawson A., Morris A. D., Struthers A. D. (2005). The epidemiology of left ventricular hypertrophy in type 2 diabetes mellitus. *Diabetologia*.

[7] Nguyen M. T., Cosson E., Valensi P., Poignard P., Nitenberg A., Pham I. (2011). Transthoracic echocardiographic abnormalities in asymptomatic diabetic patients: association with microalbuminuria and silent coronary artery disease. *Diabetes and Metabolism*.

[8] Andersson C., Gislason G. H., Weeke P. (2010). Diabetes is associated with impaired myocardial performance in patients without significant coronary artery disease. *Cardiovascular Diabetology*.

[9] Fang Z. Y., Leano R., Marwick T. H. (2004). Relationship between longitudinal and radial contractility in subclinical diabetic heart disease. *Clinical Science*.

[10] Nakai H., Takeuchi M., Nishikage T., Lang R. M., Otsuji Y. (2009). Subclinical left ventricular dysfunction in asymptomatic diabetic patients assessed by two-dimensional speckle tracking echocardiography: correlation with diabetic duration. *European Journal of Echocardiography*.

[11] Ng A. C. T., Delgado V., Bertini M. (2009). Findings from left ventricular strain and strain rate imaging in asymptomatic patients with type 2 diabetes mellitus. *The American Journal of Cardiology*.

[12] Ernande L., Bergerot C., Rietzschel E. R. (2011). Diastolic dysfunction in patients with type 2 diabetes mellitus: is it really the first marker of diabetic cardiomyopathy?. *Journal of the American Society of Echocardiography*.

[13] Seferović Mitrović J. P., Seferović P. M., Vujisić Tešić B. (2012). Predictors of diabetic cardiomyopathy in asymptomatic patients with type 2 diabetes. *International Journal of Cardiology*.

[14] Kamalesh M., Nair G. (2005). Disproportionate increase in prevalence of diabetes among patients with congestive heart failure due to systolic dysfunction. *International Journal of Cardiology*.

[15] Boonman-De Winter L. J. M., Rutten F. H., Cramer M. J. M. (2012). High prevalence of previously unknown heart failure and left ventricular dysfunction in patients with type 2 diabetes. *Diabetologia*.

[16] Parissis J. T., Rafouli-Stergiou P., Mebazaa A. (2012). Acute heart failure in patients with diabetes mellitus: clinical characteristics and predictors of in-hospital mortality. *International Journal of Cardiology*.

[17] Retnakaran R., Zinman B. (2008). Type 1 diabetes, hyperglycaemia, and the heart. *The Lancet*.

[18] Litwin S. E. (2013). Diabetes and the heart: is there objective evidence of a human diabetic cardiomyopathy?. *Diabetes*.

[19] Lang R. M., Bierig M., Devereux R. B. (2005). Recommendations for chamber quantification: a report from the American Society of Echocardiography's guidelines and standards committee and the Chamber Quantification Writing Group, developed in conjunction with the European Association of Echocardiography, a branch of the European Society of Cardiology. *Journal of the American Society of Echocardiography*.

[20] Valensi P., Pariès J., Brulport-Cerisier V. (2005). Predictive value of silent myocardial ischemia for cardiac events in diabetic patients: influence of age in a French multicenter study. *Diabetes Care*.

[21] Regan T. J., Lyons M. M., Ahmed S. S. (1977). Evidence for cardiomyopathy in familial diabetes mellitus. *Journal of Clinical Investigation*.

[22] Somaratne J. B., Whalley G. A., Poppe K. K. (2011). Screening for left ventricular hypertrophy in patients with type 2 diabetes mellitus in the community. *Cardiovascular Diabetology*.

[23] Garavaglia G. E., Messerli F. H., Nunez B. D., Schmieder R. E., Grossman E. (1988). Myocardial contractility and left ventricular function in obese patients with essential hypertension. *The American Journal of Cardiology*.

[24] Lavie C. J., Ventura H. O., Messerli F. H. (1992). Left ventricular hypertrophy: its relationship to obesity and hypertension. *Postgraduate Medicine*.

[25] Kosmala W., O'Moore-Sullivan T., Plaksej R., Przewlocka-Kosmala M., Marwick T. H. (2009). Improvement of left ventricular function by lifestyle intervention in obesity: contributions of weight loss and reduced insulin resistance. *Diabetologia*.

[26] Marwick T. H. (2008). Diabetic heart disease. *Postgraduate Medical Journal*.

[27] Zhi Y. F., Prins J. B., Marwick T. H. (2004). Diabetic cardiomyopathy: evidence, mechanisms, and therapeutic implications. *Endocrine Reviews*.

[28] Nitenberg A., Ledoux S., Valensi P., Sachs R., Attali J.-R., Antony I. (2001). Impairment of coronary microvascular dilation in response to cold pressor—induced sympathetic stimulation in type 2 diabetic patients with abnormal stress thallium imaging. *Diabetes*.

[29] Nagueh S. F., Appleton C. P., Gillebert T. C. (2009). Recommendations for the evaluation of left ventricular diastolic function by echocardiography. *European Journal of Echocardiography*.

